# A long-term survivor of repeated inguinal nodes recurrence of papillary serous adenocarcinoma of CUP: case report

**DOI:** 10.1186/1477-7800-3-22

**Published:** 2006-08-25

**Authors:** Takeshi Todoroki, Souichiro Murata, Yuji Nakagawa, Nobuhiro Ohkohchi, Yukio Morishita

**Affiliations:** 1 Department of Surgery, Institute of Clinical Medicine, University of Tsukuba, Tsukuba-Shi, 305-8575, Japan; 2 Department of Pathology, Institute of Clinical Medicine, University of Tsukuba, Tsukuba-Shi, 305-8575, Japan

## Abstract

**Background:**

Tumor spread beyond the peritoneal cavity in cases of papillary serous adenocarcinoma of the unknown primary (CUP) is a rare late event and carries a poor prognosis.

**Case presentation:**

A 71-year-old female was referred to our hospital because of a large right inguinal tumor with biopsy evidence of carcinoma as well as an elevated serum CA125 (cancer antigen 125). She underwent complete resection of the right inguinal tumor and multiple pelvic tumors, which involved the rectum, ovary and uterus. Pathological examination revealed the tumors to be metastases of a papillary serous adenocarcinoma with a psammoma body of CUP. On the 28th postoperative day, newly developed asymptomatic small left inguinal node metastases in the setting of a normal CA125 level were removed. Four and a half years after the primary resection, the CA125 level increased again and newly developed asymptomatic metastases were found in the right deep inguinal nodes and extirpated at that time. All surgical resections followed the modified FAM (5FU, Adriamycin; ADM, MMC) regimen, including protracted dairy oral administration of UFT or 5'-FDUR, Cimetidine and PSK (protein-bound polysaccharide K) as an immunomodulator or biological response modifier in conjunction with intermittent one-day continuous infusion (ADM+MMC) or intermittent single bolus injection of ADM+MMC. At present, the patient has been living in good health for almost 7 years with no evidence of relapse.

**Conclusion:**

Aggressive resection surgery followed by effective adjuvant chemotherapy is necessary for surviving long time without relapse of poorly prognostic patients with metastases outside of the abdominal cavity from peritoneal papillary serous adenocarcinomas.

## Background

Despite recent advances in diagnostic imaging and molecular pathology, CUP accounts for about 3 % of all malignant neoplasms, which represent a group of heterogeneous tumors sharing unique clinical characteristics of metastatic epithelial cancers with no identifiable origin even on autopsy [1]. The heterogeneity of CUPs in their clinical presentations, histologic types and natural histories has rendered accurate diagnosis and appropriate treatment strategies difficult. Female patients with peritoneal papillary serous carcinomatosis form a subset of patients who respond favorably to cisplatin-based chemotherapy; however, metastases outside of the peritoneal cavity are unusual and carry a dismal prognosis [2,3]. There is no standard chemotherapeutic regimen for repeated metastases outside of the peritoneal cavity in cases of peritoneal papillary serous adenocarcinomatosis [4].

Herein, we present a female patient with repeated bilateral inguinal lymph node metastases from peritoneal papillary serous adenocarcinomatosis of CUP, who is alive and well almost 7 years after the initiation of therapy involving multiple resection surgeries and unique adjuvant chemoimmunotherapy.

## Case presentation

### Present history and preoperative examination

**Figure 1:**
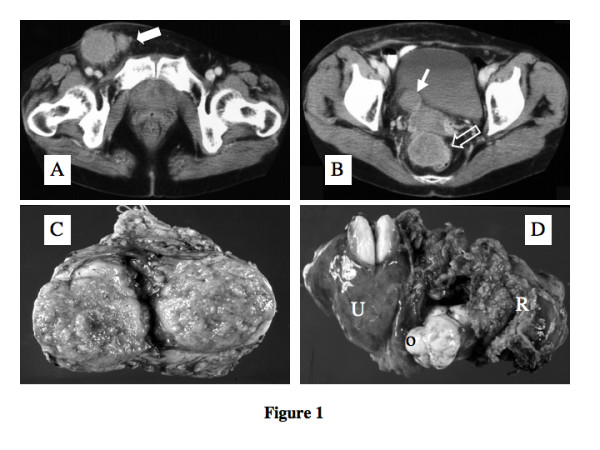
A. CT demonstrated a large lump of inguinal tumor (arrow) on the right femoral vessels. B. CT showed tumors with 3 cm in diameter locates between urinary bladder and uterus (solid arrow) and a bigger (5 cm) tumor (hallow allow), showing invasive growth to the adjacent uterus and rectum. C. Right inguinal tumor is circumscribed fairly well and measured 42 × 25 × 12 mm in size. The cut surface is swell up and reddish-white, and had hemorrhagic and necrotic foci. D. A lump of intra-pelvic tumors removed by total hysterectomy (U) with salpingo-oophorectomy and a partial resection of the rectum wall (R) at the rectosigmoid junction.

A 71-year-old female was admitted to our hospital with a large right inguinal tumor (5 cm in diameter) on June 2, 1999. She complained of inguinal mass increasing in size for the 4 months preceding admission. She had a past history of an appendectomy for acute appendicitis 16 years previous to admission. No abnormalities were found in her complete blood and biochemistry profiles. Serum carcinoembryonic antigen (CEA), carbohydrate antigen 19–9 (CA19-9), squamous cell carcinoma antigen (SCC) were all within normal limits, but CA125 was elevated to 360 U/ml at hospitalization. Ultrasonography, CT-scan and MRI demonstrated not only a right inguinal tumor, but also showed large pelvic tumors (Fig. 1A&1B). The uterus was myomatous, but no evident tumor mass was identified elsewhere by whole body CT, MRI and scintigraphy. Endoscopic examinations of the esophagus, gastrointestinal and colorectal tracts did not reveal any sign of malignant tumor. The tumor growth pattern was rather expansive into the adjacent urinary bladder, uterus and rectum. She had several asymptomatic tiny gallbladder stones. She underwent complete tumor resection of the right inguinal tumors with salpingo-oophorectomy and partial resection of the rectal wall at the recto-sigmoid junction on July 1st, 1999.

CT on the 23rd postoperative day demonstrated new small asymptomatic inguinal tumors adjacent to the left external iliac artery. The diameter of those tumors was 15 mm and 12 mm, respectively. Those tumors were extirpated completely on July 29, 1999.

### Operative procedures and findings

The right inguinal tumor, approximately 4.5 × 5 × 4 cm in diameter, was relatively well circumscribed by the right inguinal ligament, and right external iliac artery and vein, which are supplying and draining tumor blood flow. After extirpation of the inguinal tumors, the intra-pelvic tumors were removed completely by total hysterectomy with salpingo-oophorectomy and a partial resection of the rectum at the rectosigmoid junction (Fig. 1C&1D). There was no direct invasion of the tumor into the urinary bladder and there was no evidence of remaining tumor in the abdominal cavity.

### Pathologic findings and diagnosis

**Figure 2:**
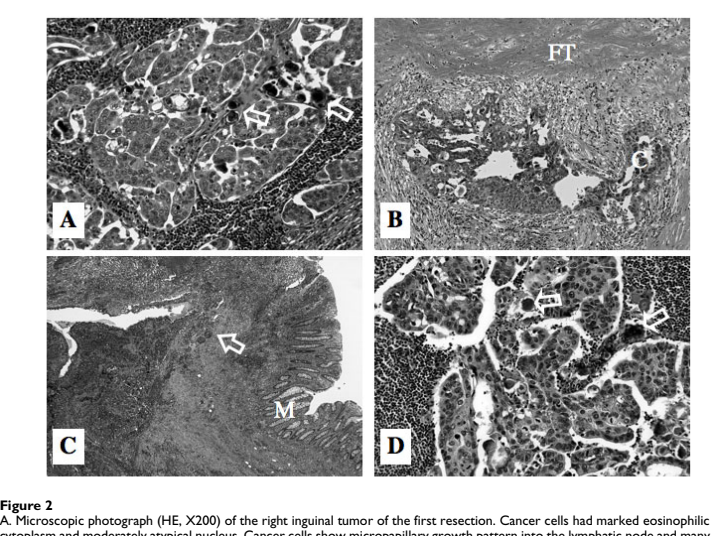
A. Microscopic photograph (HE, X200) of the right inguinal tumor of the first resection. Cancer cells had marked eosinophilic cytoplasm and moderately atypical nucleus. Cancer cells show micropapillary growth pattern into the lymphatic node and many Psammoma bodies (hallow arrow) are evident. These findings indicate metastasis of serous papillary adenocarcinoma. B. Microscopic photograph (HE, X200) of the ovarium. Almost whole ovarial tissue had been replaced by fibrothecoma (FT) without carcinoma cells (C), but tumor cells invaded from the out side of the ovary. C. Microscopic photograph (HE, X40) of the rectum. Adenocarcinoma cells infiltrated from the serosal side to the submucosal layer (hallow arrow), however, no cancer cells had reached to the mucosal layer (M). D. Microscopic photograph (HE, X200) of the right inguinal lymph node of the 3rd resection. Metastasized serous papillary adenocarcinoma cells are growing in micropapillary pattern into the lymphatic tissue similarly as Fig. 2-A. Hallow allow represents Psammoma body.

Grossly, the right inguinal tumor was elliptical and well circumscribed, measuring 42 × 25 × 12 mm in size. The cut surface of the solid tumor was swollen and reddish-white, with hemorrhagic and necrotic foci (Fig. 1C). Histologically, the tumor was encapsulated by dense fibrous tissue. The configurations of the individual tumor cells were cuboidal or polygonal and growing in a micropapillary pattern. The tumor cells had marked eosinophilic cytoplasm and moderately atypical nuclei. Many psammoma bodies [5] were recognized and these findings facilitated the diagnosis of metastatic papillary serous adenocarcinoma (Fig. 2A). Almost all of the ovarian tissue had been replaced by a fibrothecoma without carcinoma cells, but tumor cells invaded from out side of the ovary (Fig. 2B). There were 4 leiomyomas within the uterus, but no cancer cells were recognized anywhere in the uterus. On the rectum wall, carcinoma cells infiltrated from the serosal side to the submucosal layer; however, no cancer cells had reached the mucosal layer (Fig. 2C).

### Postoperative course and adjuvant therapy

Her postoperative course was uneventful, except for the early detection of the left inguinal node metastasis by CT on the 23rd postoperative day. This tumor was extirpated promptly and postoperative imaging procedures confirmed that no tumor remained anywhere in the body. Pathologic examinations revealed lymph node metastasis from a papillary serous adenocarcinoma (Fig. 2D). Preoperatively, daily oral administration of UFT (450 mg/day) Cimetidine (H_2 _receptor antagonist; 600 mg/day) and PSK (3 g/day) was started. We used Cimetidine not only as an anti-ulcer drug, but also as an immunomodulator or biological response modifier with the hope of eradicating possible microscopic residual cancer cells [6-8]. PSK was used as an immunomodulator against possible remaining adenocarcinoma cells somewhere in the body of this patient [9,10]. In order to increase the anti-tumor effect as well as decrease the toxicity, we tried intravenous single-day continuous infusion of low dose combination chemotherapy through a port implanted on the right upper chest wall with an access catheter (Groshong^®^) to the superior vena cava by way of the right subclavian vein. A portable elastomeric infusion system filled with 50 ml saline dissolution of Mitomycin C (MMC; 6 mg) and Adriamycin (ADM; l0mg) was applied every other week at an outpatient clinic. Three months after we changed from UFT to 5'-FDUR (600 mg/day) because MMC may be more effective with 5'-FDUR (due to stronger inductive activity of thymidine phosphorylase (dThdPase) in tumor tissues [11,12].

**Figure 3:**
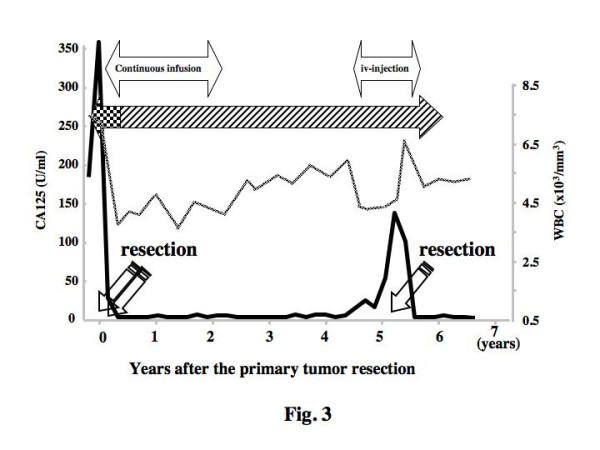
Changes of CA125 and white blood cell count after treatment. The serum level of CA125 (). The white blood cell number () Daily oral administration of UFT (450 mg/day);  or 5'-FDUR (600 mg/day);  plus Cimetidine (H_2 _receptor antagonist; 600 mg/day) and PSK (3 g/day). Continuous infusion and iv-injection; see text.

The implanted port and access catheter were removed two years later. The serum tumor markers CA-125, CEA and CA19-9 were checked every month and image diagnostic screening by CT and US were carried out every three months during 4 postoperative years, however, no suggestive signs of relapse had been found anywhere in the body. However, from 4.5 years after the last surgery, the serum level of CA-125 had gradually elevated to 140.5 U/ml and the CT scans demonstrated newly developed asymptomatic single tumor growth (25 × 20 mm) on the right deep inguinal node (Fig. 3), though no evident tumor was found anywhere else in the body. Complete tumor removal was carried out and the serum CA125 decreased to within the normal range (Fig. 3). Pathologic examination revealed that papillary serous adenocarcinoma cells were growing in the lymph node in a micropapillary pattern and many psammoma bodies were recognized (Fig. 2D). Findings of US, CT and the tumor markers have suggested no relapse of disease and oral 5'-DFUR administration was discontinued at the 6th year, but oral Cimetidine and PSK have been continued. No adverse effects were encountered at any time during the clinical course (Fig. 3) and the patient is living well, disease-free almost 7 years after the primary tumor resection.

## Discussion

Despite modern imaging technology, including Positron Emission Tomography (PET) scanning, the primary site remains unknown in patients with metastatic CUP, even on autopsy. Metastatic CUP represents a heterogeneous group in terms of clinical presentation, histology and presumably biology. Lymph node involvement, the number of metastatic organ sites, hepatic involvement, and tumor histology have been reported as significant independent prognostic factors [2]. Although the histologic features of our case presented a papillary configuration and psammoma bodies in all of the surgical specimens, no primary site was found in the body or in the ovaries. Taking into account the fact that the epithelial layer of the ovary and the peritoneum share a common origin from the coelomic epithelium embryonically and that repeated elevations of the serum CA19-9 level occurred whenever metastasis had occurred in our case, it is plausible that papillary serous carcinoma of the peritoneum is an analog with ovarian carcinoma or an extraovarian primary peritoneal carcinoma [3,14]. Clinical experiences in treating patients with inguinal nodes metastases of female peritoneal papillary serous carcinomas have been reported infrequently and there is a lack of a standard chemotherapeutic regimen for this unusual and poor prognostic disease [2,15]. Cisplatin-based chemotherapy might be a choice in our case, given that favorable response rates [3,14,15] have been reported in female peritoneal papillary serous carcinoma cases if metastases outside the peritoneal cavity have not been present. However, we chose modified FAM, because a regimen including 5-FU, ADM and MMC have shown some valuable activity in many adenocarcinomas of the gastrointestinal tract, pancreas, lung and breast, etc. Instead of 5-FU we used UFT and 5'-DFUR, which are synthesized fluoropyrimidine prodrugs of 5-FU for oral administration and have been designed to improve the delivery of 5-FU. Both are intended to increase the anti-cancer efficacy and reduce the toxicity by slow conversion of the drug to fluorouracil through the cytochrome P-450 metabolic pathway [16] in the former and by dominant delivery of fluorouracil in cancer tissues in the latter [12]. We tried reciprocal enhancement of anti-tumor effects of 5'-DFUR and MMC with anticipation of less toxicity. MMC was used not only tumor cell killing, but also as an up-regulator of dThdPase, which is an essential enzyme to convert 5'-DFUR to 5-FU located more amount than in the non-cancerous tissue [12]. Cimetidine and PSK may have favorable effects as an immunomodulator or biological response modifiers to kill some microscopic residual cancer cells and to prevent possible formation of new metastases by inhibiting cancer cell adhesion to endothelial cells [6-10].

## Conclusion

Female patients with metastases to the inguinal nodes from peritoneal papillary serous carcinomas of CUP are rare and have poor prognoses. There is no standard effective chemotherapeutic regimen for these patients. However, complete tumor removal followed by appropriate chemotherapy can lead to long-term survival even if the patient had nodal metastases beyond the abdominal cavity. Our modified FAM using protracted intermittent infusion of low dose MMC/ADM combined with oral UFT/5'-FDUR with PSK/Cimetidine may prove to be an efficient chemotherapeutic regimen with low toxicity, and which is amenable to good compliance.
